# Ischemic Stroke in Patients Under Oral Anticoagulation: The Achilles Heel of Atrial Fibrillation Management

**DOI:** 10.3390/brainsci15050454

**Published:** 2025-04-26

**Authors:** Kyriakos Dimitriadis, Nikolaos Pyrpyris, Konstantinos Aznaouridis, Gyanaranjan Nayak, Panagiotis Kanatas, Panagiotis Theofilis, Panagiotis Tsioufis, Eirini Beneki, Aggelos Papanikolaou, Christos Fragoulis, Konstantina Aggeli, Konstantinos Tsioufis

**Affiliations:** 1 First Department of Cardiology, School of Medicine, National and Kapodistrian University of Athens, Hippokration General Hospital, 115 27 Athens, Greece; npyrpyris@gmail.com (N.P.); conazna@yahoo.com (K.A.); drgyana10@gmail.com (G.N.); pkanatas@outlook.com (P.K.); panos.theofilis@hotmail.com (P.T.); e.beneki@hotmail.com (E.B.); agepap25@otenet.gr (A.P.); christosfragoulis@yahoo.com (C.F.); dina.aggeli@gmail.com (K.A.); ktsioufis@gmail.com (K.T.); 2 Department of Cardiology, Lausanne University Hospital and University of Lausanne, 1005 Lausanne, Switzerland

**Keywords:** atrial fibrillation, ischemic stroke, oral anticoagulation, vitamin K antagonists, direct oral anticoagulants, thrombosis

## Abstract

Oral anticoagulation (OAC) is essential for preventing ischemic stroke events in patients with atrial fibrillation (AF), and leads to a significant ischemic prophylaxis, when appropriately used. However, there is still a risk of experiencing stroke events, despite being under anticoagulation. Stroke despite OAC is an increasingly common diagnosis, and pathophysiologically, it can be associated with several etiologies, ranging from AF competing mechanisms to true anticoagulation failure. While the cardioembolic origin of stroke is the most frequently identified etiology, other factors also have to be considered, as there is a significance risk of coexistence. This highlights the need for thorough diagnostic testing, evaluating each stroke etiology independently, with the use of imaging, biomarker and blood tests. Treating such patients, however, is more complex, as there is still uncertainty regarding the selection of OAC post-stroke, with data showing a superiority of direct OAC (DOAC), compared to vitamin K antagonists, in recurrent ischemic stroke prevention and conflicting results regarding OAC switch. Finally, the additive value of cardiac interventions, such as left atrial appendage occlusion (LAAO), in secondary prevention of stroke, is being explored, as it could potentially lead to significant stroke risk reduction. This review, therefore, provides an updated summary of the pathophysiology, diagnostics and therapeutics of stroke under OAC, while also discussing the future direction on the Achilles heel of AF management.

## 1. Introduction

Atrial fibrillation (AF) is the commonest arrhythmia, with a prevalence of 1–2% in the general population, which is constantly increasing [1]. AF’s most dreadful complication is ischemic stroke, occurring in 10–20% of AF patients, while AF increases stroke risk up to five times [2,3]. This has led to a need for anticoagulation, with AF guidelines endorsing daily anticoagulation in patients at high thrombotic risk, using oral anticoagulants (OAC) [4,5]. Direct oral anticoagulants (DOAC) are currently the most widely selected option, with 80% of AF patients with an indication for anticoagulation being prescribed a DOAC [6], as studies have shown a reduction in bleeding events without ischemic compromise, compared to vitamin K antagonists (VKA) [7]. The use of anticoagulation is estimated to reduce stroke risk by 70%, thus providing sufficient anti-ischemic protection [8].

Despite OAC use, patients still experience stroke under OAC. This clinical entity is more common than previously thought, with an estimated prevalence ranging from 2% to 30% among trials [9,10]. Stroke under OAC implies a challenge in patient management. Despite studies show greater survival and less stroke severity in such patients [11,12], there is still a need to identify those patients that suboptimally respond to OAC treatment, in order to prevent subsequent adverse events. Therefore, we present a narrative review on the pathophysiology, diagnosis and management of stroke under OAC, following a rigorous approach through literature search and including all relevant clinical studies and major reviews on AF patients who experienced ischemic stroke while on OAC, published up to 2025. Overall, this review aims to provide an up-to-date summary of the pathophysiology behind stroke under OAC in AF, analyze diagnostic algorithms as well as therapeutic implications and provide future direction in the field.

## 2. Pathophysiology

**Figure 1:**
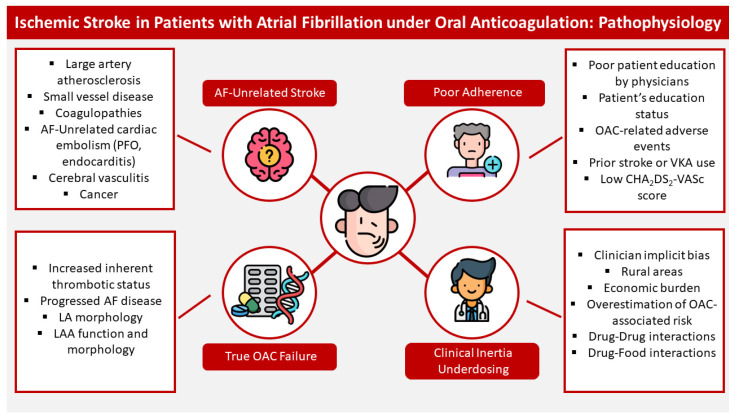
Ischemic stroke in patients under oral anticoagulation: pathophysiological relationships. This figure showcases the four recognized etiologies of stroke under OAC, which are: (i) AF-unrelated stroke, due to either cerebral vessel disease, AF-unrelated thromboembolism (thrombophilia, cancer); (ii) poor patient adherence, due to poor education of the patient on proper OAC dosing, OAC-related adverse events leading the patient to discontinuation of anticoagulation, low socioeconomic status or prior stroke; (iii) clinician inertia and underdosing; due to implicit bias, practicing in rural areas, multiple conflicting consensus documents and guidelines, drug–drug or drug–food interactions; and (iv) true OAC failure due to inherent increased thrombotic status/genetic factors of poor OAC activity and metabolism, adverse left atrial or left atrial appendage morphology, advanced disease. Abbreviations: AF: atrial fibrillation; PFO: patent foramen ovale; OAC: oral anticoagulation; LA: left atrium; LAA: left atrium appendage; VKA: vitamin K antagonist.

Numerous theories have been proposed to explain stroke despite OAC therapy, including true drug failure, poor adherence and competing mechanisms (Figure 1). However, to date there is limited evidence explicitly supporting its pathogenesis. It is plausible that a conundrum of concurrent mechanisms promotes thromboembolism; however, each mechanism is described independently in order to provide more insight regarding each pathogenetic process.

### 2.1. Non-AF Related Ischemic Stroke

Ischemic stroke is a multifaceted pathology, with a handful of etiologies beyond AF, such as large artery atherosclerosis (LAAS), small vessel disease (SVD), carotid disease, coagulopathies and hypercoagulable states (i.e., cancer) and non-AF-related cardioembolic sources, including endocarditis and open patent foramen ovale (PFO) [13]. These risk factors explain the multiple mechanisms possibly responsible for ischemic stroke, even in the presence of AF. Polymeris et al. [14] showed that up to 24% of ischemic strokes under anticoagulation are related to AF-competing mechanisms, with the majority being associated with LAAS (60.6%), followed by SVD (26.3%) and coagulopathies (5.3%). Other studies have also demonstrated that around 20% of strokes under OAC are related to competing mechanisms, with LAAS being the main contributor [15]. Moreover, when comparing patients with AF under OAC versus OAC-naïve patients, in OAC-treated patients there are significantly more competing mechanism-related strokes than in naïve patients [16]. This was also reported by Xue et al. [17], who showed that despite adequate anticoagulation, up to 42.4% of strokes are non-cardioembolic, compared to insufficient (20%) and no OAC use (23%). The rate reported by Xue et al. is larger compared to that in previous studies, which could be attributed to a smaller study size, as well as the inclusion of adequately anticoagulated patients in the cohort analysis, which could thus increase the percentage of competing mechanisms. Finally, the rate of coexistence between competing mechanisms and cardioembolic stroke is also considerable, as the RENo study showed that in 32.7% of patients, according to the ASCOD classification, there was more than one stroke etiology, with the coexistence rate of any competing mechanism and cardioembolism measured at 14.5% of all events [18].

Competing AF stroke mechanisms can also be related to recurrent ischemic strokes under OAC. These patients have an increased risk of recurrent strokes, in comparison to OAC-naïve patients [19,20]. Zietz et al. reported an adjusted hazard ratio of 2.96 for stroke recurrence in patients with LAAS and a first stroke event under OAC [15]. A similar hazard ratio was found by Ip et al. (2.84), among other risk factors such as advanced age and diabetes [21]. These results underline the importance of identifying concomitant risk factors and timely treatment in order to reduce future risk of recurrence.

### 2.2. Poor Patient Adherence

Anticoagulation within therapeutic ranges is not achieved in a large proportion of patients. Treatment with VKAs requires close monitoring of the international normalized ratio (INR), as well as proper patient training. Studies have shown that in patients with AF, time in therapeutic range (TTR) is around 60% [22]. In contrast, DOACs are considered more patient-friendly, as they do not require regular monitoring. However, a recent meta-analysis by Ozaki et al. showed that at least one in every three patients with AF under DOAC are not adherent to their prescribed medication, which was further associated with poor clinical outcomes, including a significantly increased stroke risk [23]. This increase was mostly evident in the early discontinuation phenotypes (<12 months), rather than those with declining adherence [24].

Among patients with stroke under OAC, a study by Kohlhase et al. evaluating patients with large vessel occlusion (LVO) stroke showed that more than 50% of patients with cardioembolic-originated LVO were not adherent to anticoagulation [25]. Furthermore, Tiili et al. in a similar study showed that 56% of stroke patients using warfarin and 44% of patients using DOAC reported suboptimal adherence [26]. Predictors of non-adherence include low CHA_2_DS_2_-VASc score, prior traumatic injury or fall, declining renal function, baseline antiarrhythmic use [24], as well as absence of heart failure (HF), tertiary education, smoking, use of VKA prior to stroke and prior ischemic stroke [26]. More recently, depressive disorders were also identified as a predictor for DOAC non-adherence, but not warfarin [27]. Given these results, clinicians should recognize poor adherence among AF patients and consider such etiologies when investigating stroke despite OAC, as correcting adherence with proper interventions may provide a therapeutic solution.

### 2.3. Clinical Inertia and OAC Underdosing

Clinical inertia and OAC underdosing in AF patients are well-recognized problems, with an estimated prevalence of 20% to 60% among studies [28,29]. Moreover, among stroke patients, approximately 30% may not receive an OAC prescription post-stroke [30]. Studies associate OAC underdosing with stroke incidence [31,32], greater stroke severity and mortality [33,34]; however, the data are inconsistent [35]. The presence of clinical inertia in healthcare systems is multifactorial and can be related to conflicting guideline recommendations, implicit bias [36,37], economic factors [38], area of residence [39] and overestimation of treatment-associated harm [40,41]. This phenomenon could lead to under-prescription and underdosing. A meta-analysis by Shen et al. showed that one in four AF patients received an off-label, reduced OAC dose, with rivaroxaban being more commonly underdosed [42], which resulted in increased patient mortality and cardiovascular events, compared to standard doses [43].

There are several other parameters contributing to OAC underdosing, such as drug-drug interactions (DDI). Regarding VKAs, there are numerous drug interactions related to their metabolism by CYP2C9 (antiepileptics, chemotherapeutics) [44,45], as well as the release of vitamin K from gut bacteria (antibiotics) [46]. In respect to DOAC, they are all substrates for p-glycoprotein, while apixaban and rivaroxaban are partly metabolized by the liver enzyme CYP3A4 [47]. Therefore, inducers of this cytochrome, such as antiepileptics and dexamethasone, could potentially lead to underdosing [48]; however, a clinical correlation is currently ambiguous [49,50]. Furthermore, as dabigatran requires acidity for optimal intestinal absorption, concomitant use of proton pump inhibitors may lead to suboptimal dosing, with inconclusive results regarding its incidence and significance [51]. Finally, food–drug interactions, i.e., vitamin K-containing vegetables for VKAs and St. John’s wort for DOACs, also influence OAC dosing [51].

In this context, Polymeris et al. showed that insufficient anticoagulation was the stroke etiology in 31.7% of patients. These patients were of greater age and more commonly of female sex [14]. Piciaroni et al. also showed that 45% of patients with stroke despite OAC therapy are prescribed a low-dose OAC, with 35% being off-label [18]. Both percentages were significantly different to controls without stroke. Multivariate analysis identified that low-dose OAC was not significantly related to stroke (HR:1.23; 0.93–1.65), while off-label use was (HR: 3.18; 1.95–5.85). Similarly to previous works, the latter showed patients prescribed a low dose were more frequently older, female, with worse renal function and a higher CHA_2_DS_2_-VASc score. Moreover, Xue et al. showed that a competing mechanism is infrequently found in underdosed patients, while stroke severity may be significantly greater compared to adequately anticoagulated individuals [17]. Underdosing was also more frequent with VKAs, compared to DOAC, consistent with previous results [14]. Finally, regarding the significance of DDIs in recurrent AF strokes, a study showed that the use of CYP450 or p-glycoprotein modulators was an independent predictor of subsequent strokes [21].

Finally, periprocedural interruption of OAC is a recognized reason that could be responsible for an ischemic event in a patient typically considered anticoagulated, despite being briefly unprotected. In these patients, the temporary interruption of anticoagulation facilitates a prothrombotic environment and therefore results in thrombus formation and ischemic stroke [52]. Although OAC interruption is well established in surgery and high bleeding-risk procedures, more recent studies have found no benefit of OAC continuation even in less invasive procedures, such as transcatheter aortic valve interventions [53]. Therefore, OAC interruption is a well-recognized factor leading to ischemic stroke and physicians should follow appropriate consensus guidance and employ therapeutic interventions (i.e., bridging) to minimize both the risk of ischemic and bleeding events [54].

### 2.4. True OAC Failure

Studies have shown that a cardioembolic origin of stroke is the most frequent etiology of stroke under OAC, ranging from 44.1% to 79.7% among trials [14,15,17,18]. However, the exact definition of true OAC failure is not described in consensus or guideline documents. True OAC failure consists of an ischemic stroke of cardioembolic source, under adequate anticoagulation, which cannot be explained by competing mechanisms [21]. The pathophysiology behind true OAC failure is less well understood. Regardless, its prognosis may be better than that of other etiologies [17].

Research efforts have identified several potential predictors of cardioembolism despite OAC. Such predictors include left atrial enlargement, hyperlipidemia, paroxysmal AF, history of HF and a high CHA2DS2-VASc score, mostly driven by age, diabetes mellitus and history of HF and/or stroke in the RENo study [18]; by diabetes, hyperlipidemia, prior stroke, HF, coronary artery disease and severe left atrial enlargement in the IAC study; [19] and abnormal liver function, HF, prior stroke, labile INR, combined antiplatelet/non-steroidal anti-inflammatory drug treatment and age over 75 years in the PREFER in AF study [55]. It is evident that most studies identify a high thrombogenicity risk. However, there is still uncertainty over whether risk scores such as CHA_2_DS_2_-VASc can predict ischemic strokes under OAC, as some found significant differences from naïve controls [18], while others did not [19,56].

Left atrium (LA) morphology, left atrial appendage (LAA) and AF burden can also be important parameters in patients with true OAC failure. As described, studies have found increased LA diameters in anticoagulated stroke patients compared to naïves [18,19]. This could imply that progressed AF could further promote thrombogenicity. Data supporting this hypothesis come from Ogata et al., showcasing that after stroke, LA diameter is predictive of recurrent stroke, even among patients under OAC, with an estimated HR of 1.59 (1.27–2.00) [57]. LAA function and morphology have also been associated with stroke events, as 3–15% of patients under OAC have an echocardiography-identified LAA thrombus [58,59,60], with LA volume and flow velocity being significant predictors of thrombus presence [59]. Other indicators of LAA function, such as low LAA wall velocity, have also been predictive of recurrent strokes [61]. Interestingly, LAA anatomical characteristics, such as multilobed or non-chicken wing LAA, are also associated with LAA thrombus formation in AF [62,63]; however, such investigations have not yet been pursued in stroke under OAC patients. These results indicate that LA and LAA dynamics and morphology may have a distinct pathophysiologic relationship with stroke despite adequate OAC. However, further research is required, along with genetic evaluation of true OAC failure, in order to identify exact pathogenetic mechanisms of this complex entity.

## 3. Diagnostic Implications

Identifying the responsible pathophysiological mechanism behind stroke under OAC can lead to timely implementation of secondary prevention measures and thus eliminate thromboembolic risk. Careful patient evaluation should lead to phenotype identification and patient-specific interventions, considering that more than one mechanism can be related to each event.

### 3.1. Evaluating Non-AF Related Stroke

The first step in evaluating stroke despite OAC is to examine the presence of competing mechanisms. History and physical examination are essential in guiding further diagnostic testing. Magnetic resonance imaging (MRI) of the brain can provide information regarding the potential stroke etiology. Specifically, diffusion-weighted MRI can identify LAAS and SVD phenotypes, based on lesion characteristics [64,65]. Other imaging techniques, such as magnetic resonance angiography and carotid ultrasound, can also showcase significant stenotic lesions. If LAAS and SVD are excluded, further testing evaluating uncommon etiologies may be pursued, such as coagulability testing for hypercoagulable states and coagulopathies, transesophageal echocardiogram for PFO, intracardiac tumors and endocarditis, and positron emission tomography and cerebrospinal fluid analysis for cerebral vasculitis.

### 3.2. Assessing Adherence and Underdosing

In the absence of findings from the above clinical evaluation, sufficient OAC therapy has to be assessed. The patient’s history can provide important information regarding OAC use (identify low or off-label dose), DDIs or food interactions and patient adherence. Evaluation of drug adherence and dosing must follow. Regarding VKAs, INR can provide valuable insight regarding coagulation status, with values between 2.0 and 3.0 being the desirable target, in order to prevent stroke events [66,67]. TTR is less commonly used in clinical settings, despite being extensively used in trials. Regarding DOACs, in the absence of specific tests, traditional coagulation panels such as thrombin and prothrombin time (TT and PT) can be used for evaluating dabigatran and anti-Xa agents, respectively [68,69]. More specific tests include dilute TT and ecarin-based clotting assays for dabigatran and calibrated anti-factor Xa assays for anti-Xa agents [69]. Newer techniques, such as viscoelastic testing and rotational thromboelastography (ROTEM) are also fast, point-of-care options, especially when using DOAC-specific assays, and can be well used in emergency settings [70]. Finally, adherence to DOACs can be measured in proportion to days covered (PDC) or medication possession ratio (MPR), with an effective MPR being at minimum 90% [71].

### 3.3. Diagnosing True OAC Failure

When no AF-competing mechanism or medication failure is identified, then true OAC failure can be designated as the etiology of the stroke. Currently, there are no definitive tests to confirm AF-related cardioembolism or OAC failure in the presence of sufficient OAC. TEE can be used to evaluate LA and LAA characteristics, especially the presence of thrombus, which can be suggestive, as aforementioned, of a higher stroke risk. Novel biomarkers, such as elevated 8-isoprostane levels, could serve as a thromboembolic risk prognosticator despite sufficient anticoagulation [72]; however, they have not been widely validated or evaluated and are not currently used in clinical practice.

## 4. Therapeutic Strategies

Treating stroke despite OAC is largely based on stroke etiology. An approach based on a multidisciplinary team discussion, including cardiologists, neurologists, hematologists, emergency and intensive care physicians and rehabilitation medicine, is essential for the comprehensive management of individuals with stroke under OAC. Acute treatment options, such as thrombolysis, are not shown to be harmful in meta-analyses, despite being prior anticoagulated [73,74,75]; however, recent data suggest a signal for harm [76], while currently intravenous thrombolysis is infrequently used in such patients in real-world settings [77]. Mechanical thrombectomy can also be safely performed [78,79]. Of great importance is monitoring the patient for hemorrhagic conversion. There is a lack of reporting of the rate of ischemic strokes under OAC that convert to hemorrhagic strokes in the aforementioned epidemiologic studies; however, it is known that ischemic strokes in AF patients are associated with increased hemorrhagic transformation and worse outcomes [80]. In such patients, careful clinical observation and serial imaging is necessary to avoid acute adverse events, with physicians being ready to withhold or reverse anticoagulation in case of acute hemorrhage. Following acute phase treatment, stroke risk factors should be addressed, as recurrent strokes under OAC are increasingly associated with competing mechanisms [21]. Importantly, as in all patients following a stroke event, rehabilitation programs should be discussed with the patient, aiming to enhance quality of life, focusing on improving both cognitive and physical/motion function [81,82]. When a non-AF mechanism is responsible, treatment should follow guidelines for each respective pathology. Furthermore, proper dosed OAC should be initiated before patient discharge, considering patient education on drug adherence [83]. However, available options for managing true OAC failure are less clear. Currently, there is a debate regarding whether switching OAC regimen or following the original strategy could alter future events, as well as for the role of LAA occlusion (LAAO).

### 4.1. Changing Oral Anticoagulation Strategy

It is logical to hypothesize that, as the original OAC of choice failed to prevent ischemic events, changing regimens could result in enhanced outcomes. Supporting this hypothesis, studies have shown that in clinical practice both a DOAC-to-VKA switch (45–50%) and a DOAC-to-DOAC switch (44–96%) are common strategies [84,85]. Thus, several studies have evaluated post-stroke OAC in anticoagulated patients (Table 1). Polymeris et al. showed that DOAC use post-stroke, regardless of the previous OAC, was associated with lower odds for the composite of recurrent ischemic stroke, intracerebral hemorrhage and all-cause mortality within 3 months, but switching DOAC or VKA to another DOAC or different DOAC dose or adding antiplatelets did not result in significantly altered outcomes [14]. Similar non-significant differences regarding a DOAC switch from an initial DOAC strategy were also found by other studies, regarding ischemic and bleeding events [19,56,86]. Hsieh et al. also reported that DOAC continuation, in comparison to a change to VKA, is associated with significantly fewer recurrent stroke and major adverse cardiovascular events, with dabigatran being associated with fewer events among DOAC switch categories [87]. Moreover, Ip et al. showed that switching a DOAC to VKA or another DOAC was significantly related to more recurrent strokes, as well as acute coronary syndromes with a DOAC switch, while the addition of antiplatelets showed a trend for reduction of recurrent strokes, but was found to be non-significant [21].

The aforementioned results indicate that DOAC is potentially the treatment of choice in patients with stroke under OAC, as compared to VKA; all available studies have shown better outcomes. Regarding switching DOAC regimens, most studies report similar or negative results compared to a non-altered therapy. However, the association of direct thrombin inhibitors with enhanced outcomes, compared to other Xa inhibitors, in the study by Hsieh et al.—despite being reported in a non-randomized trial which therefore may have been biased by non-addressed confounding factors—warrants further investigation, as it can be suggestive of a class-switching effect among DOAC regimens [87]. Thus, a head-to-head comparison of available DOACs is needed in this setting to guide therapeutic decisions.

### 4.2. Left Atrial Appendage Occlusion (LAAO)

LAAO aims to eliminate thrombogenesis in the LAA, which is considered the thrombus origin in 90% of cases in AF patients and has been shown to decrease stroke risk in large, randomized trials [88,89]. As described, poor LAA function and morphology, resulting in thrombus formation, can be identified in true OAC failure and thus be responsible for the ischemic event. Therefore, LAAO can be a feasible option for secondary prevention, despite being minimally utilized (1%) [14].

Regarding surgical LAAO, Whitlock et al. recently reported a 33% stroke risk reduction in patients who underwent cardiac surgery for another indication and a concomitant LAAO [90]. Additionally, 76.8% of patients continued to receive OAC during follow-up, while ischemic protection was consistent among different OAC strategies (OAC in all or some follow-ups and no OAC use) [91]. This has led to an IA recommendation for surgical LAAO in patients undergoing cardiac surgery, on top of continuing OACs, in the 2023 American guidelines on the management of AF [5].

Regarding transcatheter LAAO, several observational studies are available (Table 2). Cruz-Gonzales et al. evaluated 115 patients with stroke under OAC undergoing LAAO and found no significant differences in periprocedural success, adverse events, device thrombus or peri-device leak, compared to controls, with a 65% risk reduction of recurrent stroke and 100% reduction of bleeding events [92]. However, during follow-up, a minority of patients were receiving OACs, while the majority were taking antiplatelets. Similar results were reported by other teams [93]. Following studies proved the efficacy of combining LAAO and concomitant OAC use. Margonato et al. [94] reported high procedural success (98%) and post-intervention OAC prescription (70%), with a primary composite endpoint of all-cause death and MACE occurring in 26% during a 47.2 month follow-up. Compared to controls, no significant interaction was found in survival regarding LAAO indication. Further studies also established high procedural success, safety and efficacy of adjunctive to OAC LAAO [95,96,97].

In a comparison of patients with recurrent stroke undergoing LAAO and patients undergoing LAAO due to OAC contraindication, Aarnink et al. [98] enrolled 438 matched pairs from two multicenter registries and showed no significant difference in ischemic stroke events between the two cohorts (2.5% vs. 1.9%; HR: 1.37; 95% CI: 0.72–2.61). However, patients with recurrent stroke had a higher thromboembolic risk (HR: 1.71; 95% CI: 1.04–2.83), while also having a lower bleeding risk (HR: 0.39; 95% CI: 0.18–0.88), which highlights the different clinical profile of the two arms and therefore the need for an individualized approach. Finally, patients with stroke under OAC undergoing LAAO also had reduced mortality rates (4.3% vs. 6.9%; *p* = 0.028) when compared to typical LAAO patients, therefore documenting the safety and efficacy of LAAO in this setting for the prevention of adverse events.

Recently, Maarse et al. [99] reported the results of a combined analysis of multiple prospective registries investigating the use of LAAO in cases of recurrent stroke under anticoagulation versus standard of care. A total of 433 patients were equally matched for a follow-up period of 2 years. At the time of the follow-up, the annualized event rate of stroke was 2.8% per patient-year in patients undergoing LAAO vs. 8.9% per patient-year in the control group, while LAAO was related to a significantly lower risk of ischemic stroke (HR: 0.33; 95%CI: 0.19–0.58). Importantly, post-procedural OAC was discontinued in 290 patients (67%), while the remaining patients received adjunctively antithrombotic treatment.

The role of LAAO in patients with stroke despite OAC is promising; however, it is limited by the above studies’ observational design and small patient cohorts. This intervention could be particularly useful in patients with OAC contraindications, in whom a recurrent stroke under VKA treatment could be adequately addressed with this procedure without the need for further anticoagulation. Future randomized trials are warranted, such as the ongoing LAAOS IV trial, which will further explore the OAC+LAAO combination in such patient phenotypes. Regardless, even though device thrombus was not highly prevalent in the aforementioned studies, the optimal short-term antithrombotic strategy in these patients is also a frontier for future research efforts.

## 5. Future Directions

**Figure 2:**
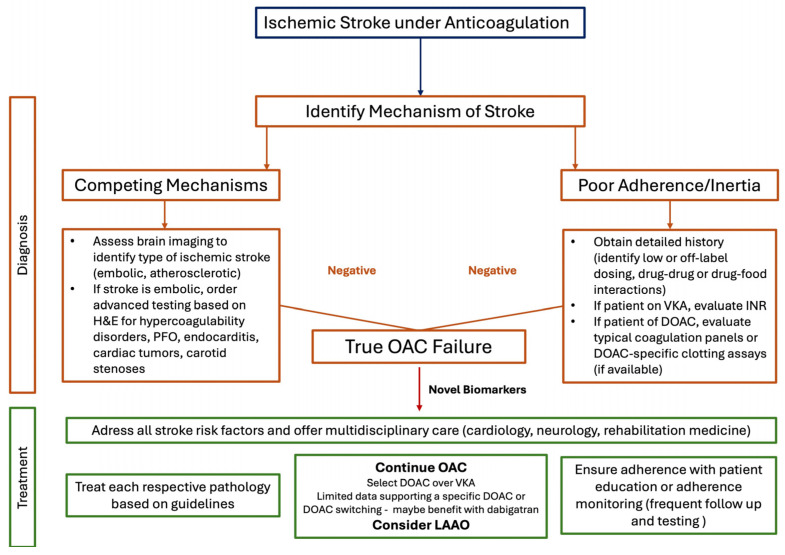
**Algorithmic approach of diagnosis and management of stroke under OAC.** In patients presenting with stroke under OAC, determining the mechanisms of stroke is the first diagnostic step. Using history and physical examination, brain imaging studies, blood tests and serum biomarkers, the clinical diagnosis of either AF-competing mechanisms or poor adherence/clinician inertia should be recognized. If the stroke cannot be attributed in these two categories, the diagnosis of true OAC failure can be made. Regarding treatment, in all patients stroke risk factors and rehabilitation after the stroke should be addressed. Then, based on the mechanism of each event, treatment should be initiated. For true OAC failure, continuing with the same DOAC is feasible, with studies needed to confirm if any agent performs better in this setting. Moreover, in patients fulfilling clinical criteria, LAAO should also be considered. Abbreviations: DOAC: direct oral anticoagulant; H&E: history and examination; INR: international normalized ratio; LAAO: left atrial appendage occlusion; OAC: oral anticoagulants; PFO: patent foramen ovale; VKA: vitamin K antagonist.

It is evident that, even though stroke under OAC is increasingly being recognized, there are still several gaps in knowledge [100]. However, as this pathology poses a relatively common clinical scenario in everyday practice, there is a need for clinical guidance in the management of these patients. Figure 2 provides an overview of a stepwise algorithm for the diagnosis and management of such patients. In the absence of robust evidence regarding the option of changing or staying with the same DOAC, the majority of studies show no difference between the two strategies. Therefore, deciding to prescribe the same DOAC is a feasible choice, while considering patient comorbidities, appropriate dosing and adherence in an individualized manner. Importantly, despite some clinicians choosing dabigatran as their agent of choice, which as aforementioned could provide an additive protection, there are still insufficient data to support changing DOACs to this agent. Thus, in practice, until further evidence is available, continuing treatment with DOAC and managing each patient in an individualized manner, based on their risk profile and comorbidities, is an essential and reasonable approach to avoid future events.

Genetic factors may also play a role in OAC failure and thus recurrent stroke despite anticoagulation. It is well established for vitamin K agonists that alterations in genes related to warfarin metabolism and activity, including CYP2C9 and VKORC1, respectively, adversely affect time in the therapeutic range and thus the levels of the maintenance dose required for each treated pathology [101,102]. Importantly, a genotype-based initiation of such agents has been shown to improve the prediction of the response to therapy and dosing, beyond the traditionally used clinical parameters [103]. For DOACs, recent studies have established new genes and respective mutations that could either increase or decrease their serum levels. Specifically, variations and single nucleotide polymorphisms of the CES1 and ABCB1 genes are responsible for alterations in the levels of dabigatran and have shown associations with mostly reduced bleeding events, but not a particular relation with increased thrombogenicity in available studies [104]. For other DOACs, similar associations have been discovered, with ambiguous results regarding the clinical importance of these findings [105]. Importantly, studies are lacking on using genotype-based guidance for DOAC treatment, as reducing the DOAC dose in variants related with bleeding could compromise ischemic protection, while there is no consensus on genetic testing or guidance prior to DOAC administration. Nevertheless, in cases of true OAC failure, clinical and research efforts should focus on more extensive genetic studies and association, as well as determining any benefit of genetic guidance and counseling for future DOAC prescription, to identify genetic predisposition to no response and suboptimal therapeutic levels and allow timely intervention and prescription of the most effective regimen in an individualized manner [105,106].

In order to better understand the management of this pathology and improve patient outcomes, future research should further evaluate the etiology and prognostic factors of this patient phenotype in order to enforce primary prevention measures aiming to decrease ischemic events. Along with research regarding pathophysiology, updates in the management and secondary prevention of such patients have great potential in altering patient outcomes. Specifically, a lot of novel therapies may have a role in reducing stroke events in such patients. Novel OACs, such as factor XIa inhibitors, that are more directed to thrombogenesis rather than hemostasis [107], may be a new therapeutic option for such patients, with phase 3 clinical trials currently ongoing in AF patients (NCT05643573). Similarly, pharmacotherapy studies are also ongoing for the acute phase of recurrent stroke, with the DOAC-IVT study further assessing the safety of intravenous thrombolysis in patients with ischemic stroke under DOAC treatment (NCT06241677). Moreover, ongoing trials assess the safety and efficacy of percutaneous LAAO on top of anticoagulation (NCT05976685) or without add-on anticoagulation (NCT05963698), including patients with recurrent stroke and in comparison to standard DOAC treatment. Furthermore, early AF ablation is currently being tested to determine potential reductions in stroke risk (NCT01288352). Other interventions, such as carotid filters in patients with high thrombogenic risk, are also being tested [108]. PFO closure is another intervention aiming to reduce stroke risk; however, its significance in AF has not been evaluated, as these patients were excluded from key trials. Further studies should assess if, by eliminating ischemic risk with PFO in AF patients, along with standard of care, there are associated reductions in stroke events. Finally, it is evident that the identification of OAC failure predictors in the coming years could expand indications for interventions such as LAAO and early AF ablation in respect to primary stroke prevention.

Finally, the pharmacoeconomic impact of stroke under anticoagulation and the respective treatment strategies also remain largely unknown. It is well established that OAC prescription, and especially DOAC, after cardioembolic ischemic stroke are cost-effective in long-term patient management [109,110]. Similarly, in comparison to OAC, in cases of OAC contraindications, LAAO is also a cost-effective alternative when compared to the standard of care, as it provides an incremental cost-effectiveness ratio and reduces average healthcare system-related costs due to reducing long-term care, which could be particularly useful in public health systems [111]. Importantly, studies show that LAAO could be more cost-effective than OAC, or even cost-saving in the long term [112,113], and therefore should be considered in suitable patient phenotypes, including patients with stroke under OAC. However, in the setting of recurrent stroke, much less research is available [114], showcasing an increase in healthcare and disability costs, compared to that in patients experiencing the first event. However, significant differences in the reporting of costs were noted between studies due to the different study designs and healthcare systems in each country, thus limiting the generalizability of these results and necessitating further studies. In particular, besides establishing clinical efficacy of different strategies (OAC continuation, LAAO), they should be also compared under a cost–benefit perspective in this population to help develop future clinical practice suggestions.

## 6. Conclusions

Stroke under OAC is a relatively common complication in anticoagulated AF patients, contributing to suboptimal patient outcomes. Identifying the etiology of stroke and recognizing true OAC failure can guide therapeutic interventions, such as post-stroke anticoagulation and potentially LAAO. Future efforts in the field are needed in order to better understand its complex pathophysiology, find OAC failure predictors and evaluate the role of novel interventions in secondary stroke prevention.

## Figures and Tables

**Table 1:** Studies evaluating post-stroke antithrombotic strategies in patients with stroke under anticoagulation.

Study	Year	Patient Population (n)	Median Age, yrs	Mean CHA2DS-VASc Score	Mean HAS-BLED Score	Prior to Stroke Anticoagulation	Post-Stroke Antiplatelet Strategy			
							Post-Stroke DOAC vs. VKA	DOAC to DOAC (Same or Switch)	VKA to DOAC	Antiplatelets
Polymeris et al. [14]	2022	Patients with AF and IS despite OAC (2946)	81 (76–86)	NR	NR	VKA (43.2%), DOAC (56.8%)	85.4% received DOAC post-stroke. DOAC use was associated with a reduction of 51% in the composite endpoint (recurrent IS, ICH and all-cause death at 3 months)	No significant outcome difference (HR 0.83, 0.54–1.27)	Significantly lower composite outcome events (HR 0.51, 0.33–0.79)	Addition of antiplatelets was not related to enhanced outcomes (HR:1.34, 0.89–2.03)
Yaghi et al. [19]	2021	Patients with AF and IS despite OAC (546) vs. no OAC (972)	77 (68–84)	5 (4–6)	NR	DOAC 50.9%	NR	Switching OAC class showed a non-significant difference in recurrent IS (HR 0.35, 0.11–1.13) This was also non-significant after cofounder adjustment (HR 0.41, 0.12–1.33)	NR	NR
Ip et al. [21]	2023	Patients with AF and IS despite OAC (2337)	DOACsame: 78.9 ± 9.8 DOACswitch: 79.1 ± 9.2 Warfarin: 76.4 ± 10.4	DOACsame: 4.6 ± 1.7 DOACswitch: 4.51. ± 7 Warfarin: 4.6 ± 1.8	DOACsame: 2.4 ± 0.9 DOACswitch: 2.3 ± 1.0 Warfarin: 2.4 ± 1.1	DOAC 100.0%	Compared with DOACsame, warfarin was associated with increased recurrent IS (HR 1.96, 1.29–3.02, *p* = 0.002)	DOAC switch was associated with more IS (HR 1.62, 1.25–2.11, *p* < 0.001) and ACS (HR 2.18, 1.29–6.67 *p* = 0.003), compared to same DOAC	NR	Antiplatelets did not reduce recurrent IS risk (HR 1.28, 0.88–1.84), ICH (HR 1.20, 0.54–2.68), ACS (HR 1.71, 0.80–3.66), or mortality (HR 1.09, 0.84–1.41)
Seiffge et al. [56]	2020	Patients with AF and IS despite OAC (1195) vs. no OAC (4119)	78 (71–84)	5 (4–6)	3 (3–4)	DOAC 13.5%, VKA 72.4%, undetermined 14.1%	OAC change was associated with decreased mortality in univariate (HR 0.5, 0.3–0.9, *p* = 0.012) but not multivariate analysis (HR 0.7, 0.4–1.2, *p* = 0.177) No differences in recurrent IS or ICH			
Paciaroni et al. [86]	2022	Patients with AF and IS despite OAC (1240)	78.9 ± 9.1	NR	NR	DOAC 100%	NR	No difference in the primary outcome (HR 1.1, 0.8–1.4), ischemic outcome (HR 1.1, 0.7–1.4) or bleeding outcome (HR 1.4, 0.7–2.5) between DOAC unchanged and changed	NR	Addition of antiplatelets to DOACs resulted in increased bleeding and ischemic events (OR, 1.7, 1.1–2.9, *p* = 0.03)
Hsieh et al. [87]	2023	Patients with AF and IS despite OAC (3579)	Warfarin: 77.9 ± 10.3 Apixaban: 78.4 ± 9.8 Dabigatran: 74.0 ± 9.5 Edoxaban: 77.8 ± 10.3 Rivaroxaban: 77.6 ± 10.0	Warfarin: 5.62 ± 1.36 Apixaban: 5.16 ± 1.33 Dabigatran: 4.97 ± 1.39 Edoxaban: 4.85 ± 1.53 Rivaroxaban: 5.44 ± 1.33	Warfarin: 3.13 ± 0.92 Apixaban: 3.08 ± 0.76 Dabigatran: 2.98 ± 0.83 Edoxaban: 2.96 ± 0.82 Rivaroxaban: 3.02 ± 0.74	NR	Compared to VKA, switching to any DOACs was associated with a 69% to 77% reduced risk of MACE Apixaban: HR 0.25, 0.16–0.39 Dabigatran: HR 0.17, 0.11–0.25 Edoxaban: HR 0.31, 0.17–0.56 Rivaroxaban: HR 0.31, 0.23–0.41 Similar reductions were present regarding the net composite outcome of any IS/MACE/ICH/SAH/death			

Abbreviations: VKA; vitamin K antagonist; DOAC: direct oral anticoagulation; AF: atrial fibrillation; IS: ischemic stroke; OAC: oral anticoagulation; HR: hazard ratio; NR: not reported; ACS: acute coronary syndrome; ICH: intracerebral hemorrhage; MACE: major adverse cardiovascular events; SAH: subarachnoid hemorrhage.

**Table 2:** Studies assessing left atrial appendage occlusion in patients with stroke under anticoagulation.

Study	Year	Patient Population (n)	Mean CHA2DS-VASc Score	Mean HAS-BLED Score	Device	Outcomes		
						Safety	Efficacy	Post LAAO OAC
Cruz-Gonzalez et al. [92]	2020	Patients with previous stroke under OAC (115) vs. other LAAO indication (932)	5.5 ± 1.5 vs. 4.3 ± 1.6	3.9 ± 1.3 vs. 3.1 ± 1.2	ACP	Similar procedural success (97%) and no difference in periprocedural complications between controls and stroke patients.	65% reduction of stroke risk in stroke patients (78% in controls), 100% reduction of bleeding risk (80% in controls). No significant differences.	Most commonly antiplatelets were prescribed, OAC was used in 8.1% of stroke and 4.3% of controls patients
Pracon et al. [93]	2022	Patients with previous stroke under OAC (39), LAAO controls (156)	5.0 (3.0–6.0) vs. 4.0 (3.0–5.0)	2.0 (1.0–3.0) vs. 3.0 (2.0–3.0), *p* = 0.006	Amplatzer or Watchman	Similar procedural success. No difference in the rates of devices used. Similar rates of device thrombosis (13.2% vs. 11.3%, *p* = 0.778).	Thromboembolic events were significantly lower in controls (10.3% vs. 1.9%, *p* = 0.031). No difference in major bleeding events (0.0% vs. 5.1%, *p* = 0.361).	Post-procedural DAPT was commonly used. OAC was initiated in 9 patients as a results of device-related thrombosis.
Margonato et al. [94]	2023	Patients with stroke under OAC (102) vs. matched LAAO controls (102)	3 ± 3 vs. 3 ± 2	NR	Amulet and Watchman	Procedural success was similar between the two cohorts (98%). No major periprocedural events occurred.	The primary composite of all-cause death, stroke, systemic embolism and major bleeding occurred in 26% of stroke patients.	68% OAC, 27% SAPT or DAPT. OAC at discharge (OR 0.29, 0.11–0.80, *p* = 0.017) was associated with the occurrence of primary endpoint.
Freixa et al. [95]	2019	Patients with stroke under OAC (22)	4.5 ± 1.3	2.6 ± 1.1	ACP or Watchman	Procedural success was 100%. No major periprocedural adverse events. 1 device-related thrombus occurred.	At median follow-up of 1.8 years only 1 stroke and 1 transient ischemic attack were reported. Risk reduction before and after LAAO was significant (2.0 ± 1.0 events vs. 0.1 ± 0.3 events; *p* < 0.01).	86.4% were under OAC therapy.
Galloo et al. [96]	2022	Patients with stroke under OAC (15)	6 ± 1.2	5 ± 1.2	Amplatzer or Watchman	Procedural success was 100% with both devices. No periprocedural complications. 1 device-related thrombus occurred.	No hemorrhagic strokes and 2 ischemic strokes occurred. At follow-up, all mortality events (3 patients) were attributed to non-atrial fibrillation related reasons.	Long-term OAC was continued in 73.3% of patients.
Masjuan et al. [97]	2019	Patients with stroke under OAC (19)	5.3 ± 1.48	1.73 ± 1.2	ACP or Amulet	Procedural success was 100%, with no periprocedural complications.	1 ischemic complication (transient ischemic attack) and 1 minor bleeding event during a mean follow-up of 17.4 months.	Combination of LAAO with OAC was ordered for all patients, VKA 10.5% and DOAC 89.3%. Aspirin was also given during the first 3 months.
Aarnink et al. [98]	2024	Patients with previous stroke under OAC (438) vs. OAC contraindication (438)	5.0 ± 1.6 vs. 4.5 ± 1.6	2.8 ± 1.3 vs. 2.3 ± 1.2	Amplatzer or Watchman	NR	No difference in ischemic stroke events (2.5% vs. 1.9%; HR: 1.37; 95% CI: 0.72–2.61). Increased thromboembolic (HR: 1.71; 95% CI: 1.04–2.83), and reduced bleeding risk (HR: 0.39; 95% CI: 0.18–0.88), in the stroke under OAC arm. Reduced mortality rates in the stroke under OAC group (4.3% vs. 6.9%; *p* = 0.028).	On discharge, most common regimens were: DOAC: 30.2% VKA: 24.5% DAPT: 33.9%.
Maarse et al. [99]	2024	Patients with previous stroke under OAC undergoing LAAO (433) vs. standard care (433)	5 (1.6) vs. 5.5 (1.4)	2.8 (1.3) vs. 3.3 (1.1)	Watchamn, Amplatzer and Lambre	Periprocedural complications occurred in 7% of patients (pericardial effusion 2.5%; access site complications 2.5%). Two in-hospital deaths occurred.	At 2 years, patients undergoing LAAO had significantly lower annualized event rates of stroke (2.8 vs. 8.9% per patient-year). Significantly lower overall risk of ischemic stroke (HR: 0.33; 95%CI: 0.19–0.58).	Post-LAAO antithrombotics: no OAC: 40%, no OAC after imaging showing complete closure: 27%, OAC: 33%.

Abbreviations: OAC: oral anticoagulation; LAAO: left atrium appendage occlusion; DAPT: dual antiplatelet therapy; SAPT: single antiplatelet therapy; ACP: Amplatzer cardiac plug; HR: hazard ratio; CI: confidence interval.

## Data Availability

As this is a review article, no new data were created.
